# Effect of a group educational intervention on rural Chinese women’s knowledge and attitudes about human papillomavirus (HPV) and HPV vaccines

**DOI:** 10.1186/s12885-015-1682-2

**Published:** 2015-10-14

**Authors:** Jing Li, Le-Ni Kang, Bayi Li, Yi Pang, Rong Huang, You-Lin Qiao

**Affiliations:** 1 Department of Occupational and Environmental Health, West China School of Public Health, Sichuan University, Chengdu, 610041 China; 2 National Office for Maternal and Child Health Surveillance of China, West China Second University Hospital, Sichuan University, Chengdu, 610041 China; 3 Department of Medical Services, Yangcheng Maternal and Child Health Hospital, Yangcheng, 048100 China; 4 The prevention and control section for STD & AIDS, Center for disease control and prevention of Jinjiang, Chengdu, 610021 China; 5 Department of Human Resource, The People‘s Hospital of Gansu Province, Lanzhou, 730060 China; 6 Department of Cancer Epidemiology, Cancer Institute/Hospital, Chinese Academy of Medical Sciences & Peking Union Medical College, Beijing, 100021 China

**Keywords:** HPV awareness, Knowledge, Chinese women, Vaccine, Educational intervention, Change

## Abstract

**Background:**

Statistics regarding HPV prevalence and cervical cancer rates in rural China are high, however, low levels of HPV awareness and HPV-related knowledge pose a great challenge for cervical cancer control.

**Methods:**

The authors conducted an educational intervention study, which was embedded in a cervical cancer screening project in Yangcheng county, Shanxi Province, China from October to December, 2011 and was designed to assess the short-term effectiveness of a hospital-based, brief, HPV-focused session on rural Chinese women’s knowledge and attitudes. Student’s *t*-test was used when comparing quantitative variables. Chi-squared test or Fisher’s exact tests was used when comparing qualitative variables.

**Results:**

We found that following the intervention, significant increases were detected in awareness regarding HPV (5.9 % vs. 59 %, *p* < 0.001) and cervical cancer (63.0 % vs. 89.2 %, *p* < 0.001). Changes were also observed regarding women’s intention to vaccinate themselves (82.0 % vs. 89.0 %, *p* = 0.001) and their daughters (82.9 % vs. 88.0 %, *p* = 0.011), although the impact was more modest compared with the impact on change of awareness. Among women who were aware of HPV, 60.3 % knew that cervical cancer is related to HPV, while only 5.0 % knew the relationship between HPV and genital warts after the educational intervention.

**Conclusions:**

Educational campaigns, particularly those targeting women with limited education and poor access to public media or other educational channels are needed to improve knowledge regarding HPV in the general population.

## Background

Shanxi province locates at the west of Taihang Mountains, with 11 prefecture-level cities, 23 municipal districts, 11 county-level cities and 85 counties. By the end of 2012, the registered permanent resident population of Shanxi province had reached 36.1 million. Yangcheng county locates at the southeast of Shanxi province, with approximately 0.4 million population, of which nearly 60 % live in the rural areas [1]. It is reported that Yangcheng county suffers high cervical cancer incidence (81.0 /10^5^) [2] and mortality (11.2/10^5^) [3] due to lack of education, poverty, and inability to pay for health care [4]. A pooled analysis also suggested that rural women were more than twofold likely to be infected by high risk HPV than urban women in China [5]. With an estimated 500 million women in rural China, the public health challenge to prevent cervical cancer is substantial. The Chinese government has put the health of rural women firmly on their domestic agenda. From 2009 to 2011, free cervical cancer screening examinations were made accessible to 10 million rural Chinese women [6].

Studies have clearly illustrated that lack of knowledge about HPV and low levels of understanding of HPV vaccination have direct implications for women’s participation in cervical screening [7, 8]. Despite this, the importance of health education as an integral part of primary prevention for cervical cancer is often ignored [9]. Resources focused on educational campaigns to increase women’s knowledge and awareness of HPV and its vaccines may impact the success of screening programs.

A previous multi-center cross-sectional study reported that only 15.5 % of women interviewed reported to have ever heard of HPV in China. And the proportion among rural women was only 9.3 % [10]. The study also found that hospital based lectures and suggestions from doctors and nurses played a significant role in influencing decisions about being vaccinated.

As no previous study has been conducted to evaluate a way of changing the ‘low HPV awareness’ in China, our study sought to assess the short-term effectiveness of a hospital-based, local health provider oriented educational intervention on rural women’s knowledge and attitudes towards the prevention of cervical cancer and HPV infection immediately following the group education as an intervention.

If this intervention approves to be effective in raising the awareness of HPV and cervical cancer prevention among women, an increased attendance to cervical cancer screening might be achieved when such education is widely disseminated especially through the existed educational system led by China CDC. Also, it might help enhance the HPV vaccination uptake among Chinese women in the future when HPV vaccines become available.

## Methods

### Study design and population

This study was a questionnaire-based, cross-sectional study conducted from October to December, 2011, and it was embedded in a project called “the Low-Cost Molecular Cervical Cancer Screening Study (LCMCCSS)” and was funded by the Bill & Melinda Gates Foundation. It was conducted collaboratively by Program for Appropriate Technology in Health (PATH) and the Cancer Institute of the Chinese Academy of Medical Sciences (CICAMS) after approval by the Institutional Review Boards of both organizations.

Stratified randomized cluster sampling was used in one of the cervical cancer high-risk provinces of China, Shanxi province. Initially, a cervical cancer high-risk county from Shanxi, the Yangcheng county, was selected. Two high risk communities (Baisang Xiang and Dingdian Town) from Yangcheng county were then targeted based on the estimated numbers of women registered on the Shanxi permanent residency registry. Twenty-two villages as clusters were then randomly selected from a total of 38 villages to achieve a target population of 3,000. All non-pregnant women aged 25–65 years without a history of Cervical Intraepithelial Neoplasia (CIN), cervical cancer, or hysterectomy and who were mentally and physically competent to provide written informed consent were eligible for the ‘LCMCCSS’ screening study regardless of their marital status.

**Fig. 1:**
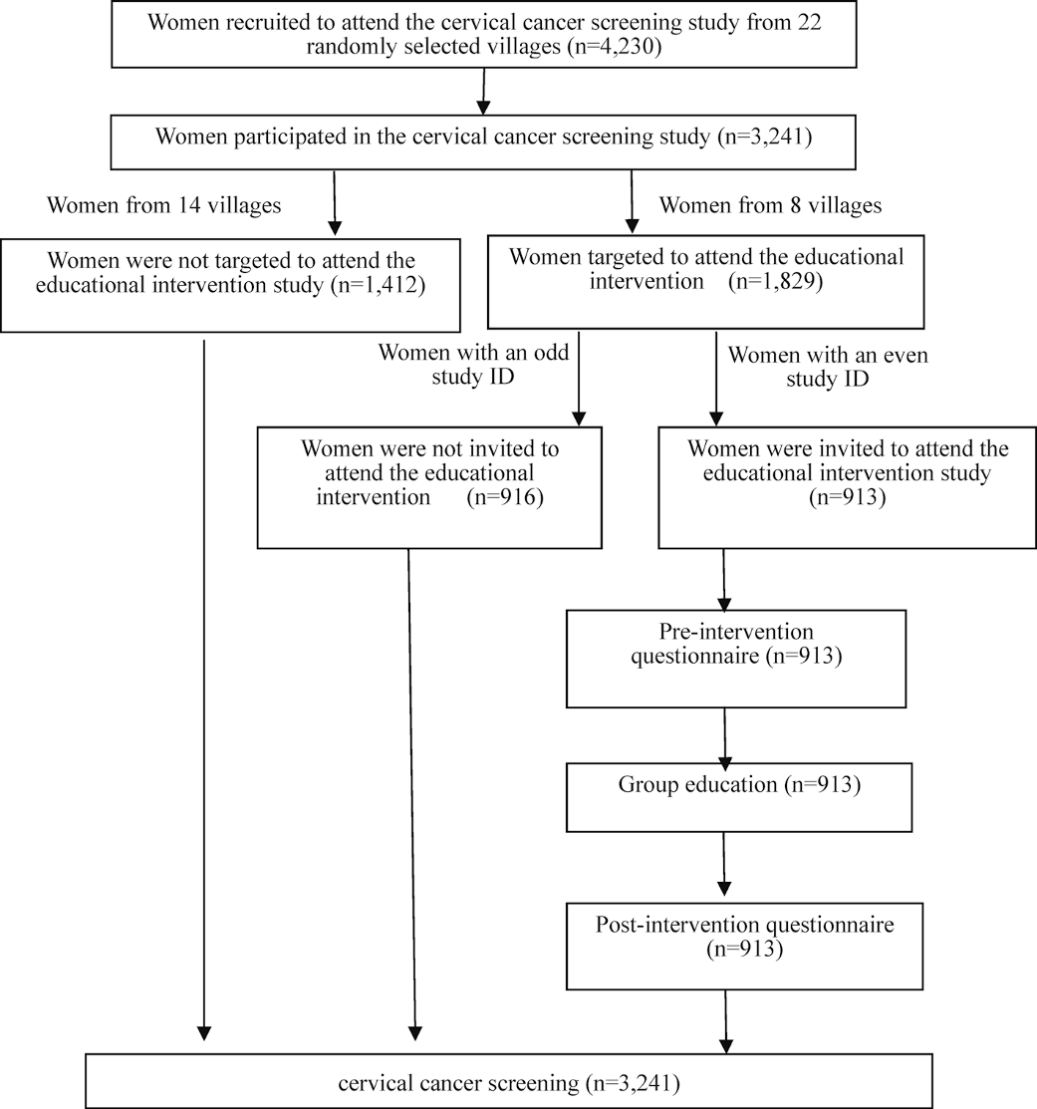
Study population

Among the 22 villages that were targeted for cervical cancer screening, 8 as clusters were randomly selected for an extra educational intervention. Among women from the 8 villages, those who had an even last digit in their randomly allocated study ID were invited to attend our educational intervention study (Fig. 1).

### Participant recruitment procedure

For the cervical cancer screening study: All recruitment was done by village doctors. They were trained by investigators from CICAMS prior to the initiation of the study. After the training, a list of targeted women and their basic information (e.g. age, living address, contact) was provided by the local permanent resident registration system. The trained village doctors then dropped house-to-house visits to women according to the list. During the house visits, the inclusion and exclusion criteria as well as the potential benefits and risks were clearly explained to women. For those who verbally agreed to participate, village doctors made a screening appointment for them.

For the educational intervention study: Recruitment was done on the same day when women attending cervical cancer screening at the screening site (the local hospital) by investigators from CICAMS. Free transportation and cervical cancer screening were offered to women who were willing to participate in the educational intervention study.

### Informed consent

Women who were willing to attend the educational intervention study provided informed consents on a voluntary basis. During the process, the words ‘HPV’ and ‘cervical cancer’ were not mentioned in order to avoid possible contaminations to the educational intervention. Instead, women were carefully explained by the investigators that they would be asked about some facts of an organ-specific female cancer, its etiology and methods to prevention. Questions would be asked both before and after a PowerPoint (PPT) oriented health education by trained interviewers.

### Pre-intervention HPV questionnaire

Immediately after providing the informed consent participants were administered a ‘Pre-intervention HPV questionnaire’ in independent rooms by trained female nurses aged 26–40 years. Questions on ①: Have you ever heard of HPV? ②: Have you ever heard of cervical cancer? ③: Do you have daughter (s)? ④: If prophylactic HPV vaccines were available, would you be willing to vaccinate your daughter? ⑤: If prophylactic HPV vaccines are available, would you be willing to vaccinate yourself? (only for women aged ≤ 45 years) were asked in this section.

When asked, women only need to answer ‘Yes’ or ‘No’. The word ‘prophylactic’ was also explained to women by the trained nurses in a very common sense way. For example, it was explained as : if you get vaccinated, you will not been infected by some types of the virus (HPV) that cause cervical cancer, like how HBV vaccines work to prevent hepatitis B infection.

### Group education

After administration of the ‘Pre-intervention HPV questionnaire’, women were convened in a meeting room in the hospital and an educational lecture was provided by a trained local doctor in local dialect by using a standardized PPT. During the lecture, women were taught basic concepts regarding ① What is cervical cancer, how does it threaten women’s health and lives;② What is HPV and its relation to cervical cancer;③ What kind of screening tests are available for the secondary prevention of cervical cancer;④ The current status of prophylactic vaccines for the primary prevention from cervical cancer. The lecture was given to 25–30 women per time by the same trained doctor, and lasted for about 40 min. Two group educations were given each day, one was in the morning and the other was in the afternoon.

### Post-intervention HPV questionnaire

After the group education, it takes a short term (about 5 min) to re-allocate women to the same interview room and they were interviewed again by the same trained nurses in a confidential setting about cervical cancer, HPV and the related preventions. In addition to questions asked in the pre-intervention questionnaire, more specific information was collected using the pre-tested questionnaire administered in the earlier study [8].

### Other questionnaires and clinical procedures

Prior to the screening and after informed consent for the screening study, the participants were administered a risk factor questionnaire, which contained not only the socio-demographics but also some risk factors related to HPV infection and cervical cancer (e.g. age at sexual debut, No. of sexual partner (s) etc.). Women then received gynecological examinations in the study clinic situated in their residential district. Cervical and vaginal specimens were collected by doctors from women undergoing routine examinations.

### Data collection and quality control

Two data input clerks in each site were recruited to double-enter data from the paper to computer-based database (FoxPro) independently after training. All finished double-entry databases were sent to CICAMS for validation by running EpiData. Any inconsistency found by CICAMS between the two databases was reported to the local clerks for adjudication until the databases agreed. As final check, one database was chosen and underwent a final consistency check. Logic errors (e.g. a woman reported had never heard of HPV yet knew the link between HPV and cervical cancer) were again reported back to the local sites, and the local collaborators contacted the participants using the contact information provided in the risk factor questionnaires. After checking with the participants, the local staffs sent the revised database back to CICAMS for final analysis. This procedure was strictly followed as previously described [10].

### Statistical analyses

Basic descriptive statistics and frequency calculations were performed on all demographic variables. Student’s *t*-test was used when comparing quantitative variables. Chi-squared test or Fisher’s exact tests was used when comparing qualitative variables. SAS statistical software version 9.1 (SAS Institute Inc., Cary, NC, USA) was used to analyze the data. Statistical significance was assessed by two-tailed tests with α level of 0.05.

## Results

### Participants profile

Of the 4,230 women that were recruited to attend the cervical cancer screening, 3,241 women came with a compliance of 80.9 %. Among them, 1,829 were from the 8 villages selected for educational intervention. Among the 1,829 women, 913 women with randomly allocated even ID numbers were recruited to attend the educational intervention study and all agreed.

The mean age of participants was 42.3 years (s.d. =8.7 years), with the youngest of 25 years and the oldest of 64 years. All participants were ethnic Han. Table 1 reports the distribution of intervention and non-intervention groups. Differences regarding the distribution of age, education, occupation, alcohol drinking, age at sexual debut, age at first pregnancy and No. of live birth were seen between the two groups.

**Table 1:** The distribution of sociodemographics of intervention and non-intervention participants (*N* = 913)

Variable		Intervention group(*N* = 913)		Non-Intervention group(*N* = 2,328)		*p*
		*n*	%	*n*	%	
Age(yrs)	25 ~ 34	171	18.7	304	13.0	**<0.001**
	35 ~ 44	372	40.8	866	37.2	
	45 ~ 54	277	30.3	707	30.4	
	≥55	93	10.2	451	19.4	
Marital status	Married	891	97.6	2252	96.7	0.201
	Single or others	22	2.4	76	3.3	
Education	Illiteracy	42	4.6	147	6.3	**<0.001**
	Primary school	277	30.3	850	36.5	
	Middle school	472	51.7	1052	45.2	
	High school and above	122	13.4	279	12.0	
Occupation	Worker	30	3.3	40	1.7	**0.003**
	Farmer	788	86.3	2105	90.4	
	White collar/Professionals	63	6.9	116	5.0	
	Others	32	3.5	67	2.9	
Smoking history	Never	912	99.9	2323	99.8	0.531
	Ever/Current	1	0.1	5	0.2	
Alcohol drinking history	Never	846	92.7	2097	90.1	**0.022**
	Occasionally or others	67	7.3	231	9.9	
Disease History	Trichomonas	102	11.2	257	11.1	0.914
	Fungus infection	89	9.8	192	8.2	0.172
	Urinary Tract Infection	56	6.1	184	7.9	0.083
	Cervitics	10	1.1	96	4.1	**<0.001**
	None	701	76.8	1740	74.7	0.226
Age at sexual debut (yrs)	<20	139	15.2	453	19.4	**0.013**
	20	179	19.6	488	21.0	
	21	199	21.8	484	20.8	
	≥22	396	43.4	903	38.8	
No. of sexual partners	1	739	80.9	1948	83.7	0.059
	2	141	15.5	287	12.3	
	≥3	33	3.6	93	4.0	
Age at first pregnancy (yrs)^a^	<21	179	19.8	512	22.2	**0.005**
	21	133	14.7	433	18.8	
	22	184	20.3	444	19.2	
	≥23	409	45.2	919	39.8	
No. of live birth^a^	0	8	0.9	12	0.5	**0.005**
	1	273	30.1	577	25.0	
	≥2	624	69.0	1719	74.5	

^a^905 women had pregnancy history among the intervention participants

Boldface reflects statistical significance

Most women (97.6 %) from the intervention group were married and the majority (82.0 %) had only primary school or middle school education. Few women smoked (0.1 %) or drank alcohol (7.3 %). Trichomonas (11.2 %), fungal infection (9.8%), and urinary tract infection (6.1 %) were the most common self-reported previous genital infections. The average age of sexual debut was 21.1 years (s.d. =1.8 years) and the age of sexual debut for 43.4 % (*n* = 396) of participants was 22 years or older. Most women reported only having one sexual partner in their lifetime (80.9 %). Most women (69.0 %) had two or more live births. Compared with the non-intervention group, the intervention group had more women from younger age group (25–34 years) and had a higher proportion of receiving middle school education (Table 1).

### HPV awareness and attitudes about HPV vaccine before education

Before the group education, very few (5.9 %) women reported having ever heard of HPV. Women with >9 years of education were significantly more likely (22.4 %) to have heard of HPV than those with ≤9 years of education (2.9 %) (*P* <0.001). Age did not influence women’s awareness of HPV (Table 2). By contrast, 63.0 % of the women in the study had heard of cervical cancer. Both more educated women (>9 years) and younger women (≤45 years) were more likely to be aware of cervical cancer (Table 2). The majority of women were willing to vaccinate their daughter(s) (82.2 %) or themselves (80.3 %) before education at baseline (Table 2).

**Table 2:** Women’s awareness and attitudes before intervention by education level and age

Item		Years of Education			Age		
	All	≤9 years	>9 years		≤45 years	>45 years	
	*n* (%)	*n* (%)	*n* (%)	*P*	*n* (%)	*n* (%)	*P*
Have you ever heard of HPV?(*N* = 913)							
Yes	54 (5.9)	22 (2.9)	32 (22.4)	**<0.001** ^b^	40 (6.8)	14 (4.3)	0.114^b^
No	859 (94.1)	748 (97.1)	111 (77.6)		545 (93.2)	314 (95.7)	
Total	913	770	143		585	328	
Have you ever heard of cervical cancer?(*N* = 913)							
Yes	575 (63.0)	445 (57.8)	130 (90.9)	**<0.001** ^b^	416 (71.1)	159 (48.5)	**<0.001** ^b^
No	338 (37.0)	325 (42.2)	13 (9.1)		169 (28.9)	169 (51.5)	
Total	913	770	143		585	328	
If there are prophylactic vaccines, are you willing to vaccinate your daughter?(*N* = 636*)							
Yes	523 (82.2)	453 (81.5)	70 (87.5)	0.241^b^	323 (83.7)	200 (80.0)	0.229^b^
No	108 (17.0)	98 (17.6)	10 (12.5)		60 (15.5)	48 (19.2)	
Missing	5 (0.8)	5 (0.9)	0 (0.0)		3 (0.8)	2 (0.8)	
Total	636	556	80		386	250	
If there are prophylactic vaccines, are your willing to vaccinate yourself?(*N* = 585**)							
Yes	470 (80.3)	394 (79.6)	76 (84.5)	0.249^b^	-	-	-
No	103 (17.6)	91 (18.4)	12 (13.3)		-	-	
Missing	12 (2.1)	10 (2.0)	2 (2.2)				
Total	585	495	90		-	-	

*Only answered by women with daughter(s) **Only answered by women aged ≤45 years

Boldface reflects statistical significance

^b^*χ2* test

### HPV knowledge after education

After the group education, HPV awareness increased to 59.0 % (539/913). Specific knowledge about the risk of HPV, its link between cervical cancer etc. is presented in Table 3. Among women who had ever heard of HPV (*n* = 539), 60.3 % (365/539) knew that HPV is related to cervical cancer with a higher proportion (69.7 %) among more educated women compared with less educated women (58.0 %). Younger women (≤45 years) were more likely (65.7 %) to know the relationship between HPV and cervical cancer than women >45 years (47.2 %). Only 5.0 % (30/539) of women knew that HPV is associated with genital warts, with a higher proportion (8.2 %) among more educated women compared with less educated women (4.1 %), and no significant difference was observed between the two age groups (Table 3).

**Table 3:** Women’s knowledge about HPV and cervical cancer after intervention by education levels and age

Item		Years of Education			Age		
	All	≤9 years	>9 years		≤45 years	>45 years	
	*n* (%)	*n* (%)	*n* (%)	*P*	*n* (%)	*n* (%)	*P*
Have you ever heard of HPV(*N* = 913)							
Yes	539 (59.0)	437 (56.8)	102 (71.3)	**0.001** ^b^	386 (66.0)	153 (46.6)	**<0.001** ^b^
No	374 (41.0)	333 (43.2)	41 (28.7)		199 (34.0)	175 (53.4)	
Total	913	770	143		585	328	
When HPV is mentioned, you will firstly think of(*N* = 539)							
STD	58 (9.6)	44 (9.1)	14 (11.4)	**<0.001** ^c^	41 (9.6)	17 (9.6)	**<0.001** ^b^
Genital Warts	30 (5.0)	20 (4.1)	10 (8.2)		21 (4.9)	9 (5.1)	
Cervical cancer	365 (60.3)	280 (58.0)	85 (69.7)		282 (65.7)	83 (47.2)	
AIDS	23 (3.8)	19 (3.9)	4 (3.3)		16 (3.7)	7 (4.0)	
Have no idea about HPV	129 (21.3)	120 (24.9)	9 (7.4)		69 (16.1)	60 (34.1)	
Total	605	483	122		429	176	
Have you ever heard of cervical cancer(*N* = 913)							
Yes	814 (89.2)	676 (87.8)	138 (96.5)	**0.011** ^b^	532 (92.8)	282 (82.9)	**<0.001** ^b^
No	99 (10.8)	94 (12.2)	5 (3.5)		41 (7.2)	58 (17.1)	
Total	913	770	143		573	340	
Do you know that cervical cancer is early detectable and treatable? (*N* = 814)							
Yes	646 (79.4)	518 (76.6)	128 (92.8)	**<0.001** ^b^	442 (81.4)	204 (75.3)	**0.042** ^b^
No	168 (20.6)	158 (23.4)	10 (7.3)		101 (18.6)	67 (24.7)	
Total	814	676	138		543	271	

^b^*χ2* test, ^c^Fisher’s exact test

Boldface reflects statistical significance

Among women who had ever heard of cervical cancer (*n* = 814), 79.4 % (646/814) knew that cervical cancer is early detectable and treatable. Knowledge rates were higher among those with higher education level and younger age (*P* <0.05) (Table 3).

### Attitudes about HPV vaccines after education

Table 4 presents reasons for being willing and unwilling to be vaccinated after education. The majority (88.8 %; 513/578) of participants were willing to be vaccinated. Fear of getting cervical cancer without vaccination (43.8 %; 322/513), benefits to themselves (26.9 %; 198/513) and concern about having been infected with HPV (21.9 %; 161/513) were the primary reasons for women to be vaccinated. Education level did not change the reasons for women to be vaccinated (Table 4). Doubts regarding the efficacy of HPV vaccine (34.3 %; 23/65) were the major reason for unwillingness to get vaccinated, and women with >9 years of education were more likely to choose this as a reason (Table 4).

**Table 4:** Women’s attitudes about HPV vaccines after intervention by education levels

Item	To vaccinate themselves*				To vaccinate their daughter (s)			
	All	Education	Education		All	Education	Education	
		≤9 years	>9 years			≤9 years	>9 years	
	*n* (%)	*n* (%)	*n* (%)	*P*	*n* (%)	*n* (%)	*n* (%)	*P*
(1)Reasons for willing to vaccinate themselves (n = 513)					To vaccinate their daugher(s) (n = 560)			
Can Benefit myself/my daughter(s)	198 (26.9)	153 (25.2)	45 (34.9)	0.110^b^	231 (28.6)	188 (27.2)	43 (36.4)	0.187^c^
Fear of HPV infection	161 (21.9)	132 (21.7)	29 (22.5)		187 (23.1)	160 (23.2)	27 (22.9)	
Fear of having CC	322 (43.8)	276 (45.5)	46 (35.6)		334 (41.3)	296 (42.8)	38 (32.2)	
Fear of having genital warts	51 (6.9)	42 (6.9)	9 (7.0)		55 (6.8)	45 (6.5)	10 (8.5)	
**Total**	736	607	129		809	691	118	
(2)Reasons for unwilling to vaccinate themselves(n = 65)					To vaccinate their daugher(s)(n = 76)			
I don’t have risk	19 (28.4)	17 (28.8)	2 (25.0)	**0.004** ^c^	26 (31.8)	24 (32.9)	2 (20.0)	0.136^c^
Don’t think the vaccination works	23 (34.3)	23 (39.0)	0 (0.0)		24 (28.2)	23 (31.5)	1 (10.0)	
It hasn’t been widely used	7 (10.4)	4 (6.8)	3 (37.5)		10 (11.8)	7 (9.6)	3 (30.0)	
Vaccination causes risks	5 (7.5)	3 (5.1)	2 (25.0)		6 (7.1)	5 (6.8)	1 (10.0)	
Doubts on the resource	7 (10.4)	7 (11.8)	0 (0.0)		6 (8.2)	4 (5.5)	2 (20.0)	
No answer**	6 (9.0)	5 (8.5)	1 (12.5)		11 (12.9)	10 (13.7)	1 (10.0)	
**Total**	67	59	8		83	73	10	

^b^*χ2* test, ^c^Fisher’s exact test

*585 women were aged ≤45 years, 7 were excluded from the analysis due to lack of information on intent to vaccinate themselves

**Women that did not answer the question were not included in the analysis

Boldface reflects statistical significance

Six hundred and thirty six (69.7 %) women reported that they had daughters. Among them, 88.1%(560/636) were willing to vaccinate their daughters, 11.9 % (76/636) were unwilling, mainly because they do not think their daughters are at risk (31.8 %; 26/76), followed by doubt regarding the vaccine’s efficacy (28.2 %; 24/76). Education level did not influence the reasons for willingness or unwillingness to vaccinate their daughters (Table 4).

### Effect of education on women’s awareness and attitudes

Table 5 presents the responses to questions related to HPV and its vaccines before and after the group education. When women were asked if they had ever heard of HPV, a significant change (5.9 % vs. 59.0 %, *P* < 0.001) in women’s awareness was observed following the educational intervention. When asked about their awareness of cervical cancer, the proportion of ‘yes’ answers also increased significantly (63.0 % v. 89.2 %, *P* < 0.001). When questioned about their intention of being vaccinated or vaccinating their daughters, the proportion of women with positive responses increased substantially.

**Table 5:** Changes in awareness and attitudes towards HPV and its vaccines

	Pre-Intervention		Post -Intervention		Change	*P* value
	Count	Percent (%)	Count	Percent (%)	Percentage point difference	
Have you ever heard of HPV (*n* = 913)						
Yes	54	5.9	539	59.0	53.1	**<0.001** ^b^
No	859	94.1	374	41.0		
Have you ever heard of cervical cancer (*n* = 913)						
Yes	575	63.0	814	89.2	26.2	**<0.001** ^b^
No	338	37.0	99	10.8		
Are you willing to vaccinate your daughter(s)(*n* = 631**)						
Yes	523	82.9	555	88.0	5.1	**0.011** ^b^
No	108	17.1	76	12.0		
Are you willing to vaccinate yourself(*n* = 571***)						
Yes	468	82.0	508	89.0	7.0	**0.001** ^b^
No	103	18.0	63	11.0		

^b^ *χ*^*2*^ test

**5 women were excluded from the analysis due to lack of pre-educational information

***14 women were excluded from the analysis. 12 were due to lack of pre-educational information and 2 were due to lack of post-educational information

Boldface reflects statistical significance

## Discussion

This study investigated rural Chinese women’s baseline HPV knowledge and their attitudes toward HPV vaccines. It is the first study in China to evaluate the short-term effectiveness of a hospital based group education to change local women’s knowledge, beliefs and attitudes towards the prevention of HPV infection and cervical cancer.

Based on the most recent WHO position paper on HPV vaccines, the recommended primary target population are young females of 9–13 years, and the secondary target population are older adolescent females or young women. There is no defined up limit of age been vaccinated [11]. In addition, there were two peaks of HPV infection among Chinese women, one is around 20 years and the other is around 45 years old [12]. It is particular important to educate and try to understand the knowledge and attitudes of women in older age group, which was covered by our study population. This may also contributes to the future catch-up doses for elder women.

Results confirm that women living in rural China have very limited awareness of HPV and its related diseases [10, 13–16], however, no previous studies have examined how this ‘low awareness’ can be changed. The latter is especially important given HPV infection is more prevalent among women living in rural areas of China [5] and cervical cancer rates are over represented [17, 18]. Although the Chinese government has initiated the first step in controlling cervical cancer in rural China [5], lack of knowledge of risk factors may become the barrier to utilization of this service [19]. As well, poor understanding of the risks and consequences of HPV infection has been reported as a potential barrier to the wide implementation of HPV vaccination [20–23]. Therefore an intervention to increase women’s knowledge about HPV and cervical cancer is an essential component of any comprehensive cervical cancer control program.

Based on previous findings that hospital based lectures and suggestions from doctors and nurses were important in influencing decisions about being vaccinated in both rural and metropolitan areas [10], we evaluated the short-term effectiveness of a hospital based local health provider oriented lecture in changing local women’s awareness and attitudes toward HPV and its vaccines. In this present study, we found that women’s awareness both on HPV and cervical cancer changed notably especially regarding HPV immediately following the group education. Although there is no comparative data available within China, several studies from outside of China have investigated the effectiveness of educational interventions of varying duration, using various methods aiming at similar goals and found that education intervention was associated with increased HPV-related knowledge [24–28]. This study confirms that the short-term effectiveness of a one-time brief group education intervention provided in a hospital environment appears to be valid in raising awareness among Chinese women from rural China, where the majority of women have less than 9 years of education (middle school).

Immediately after the educational intervention, women’s awareness of ever heard of HPV increased from 5.9 % to 59 %, however, 41 % of them still reported that they had never heard of HPV. The reason was possibly because that 86.6 % of women in this population received middle school or less education, suggesting that they did not have a chance to learn English. In our intervention, HPV was expressed in both English as ‘HPV’ and in Chinese as ‘Ren Ru Tou Liu Bing Du’, however, in our questionnaire, it was written only in English and asked by using the English term. This may result in the situation that women were unaware of the English term ‘HPV’ even immediately after the intervention. This should also be considered in the future studies and the question should be asked by using both the English term as well as the Chinese term.

Another possibility might be that women at this education level were not very interested in a PPT oriented education, and some of them were absent minded during the intervention, a more easy and interesting way to conduct such intervention should be considered among women that were less educated.

Compared with the very low baseline awareness of HPV, the acceptability of vaccination seemed to be high at baseline. Although increases were also observed in women’s attitudes toward HPV vaccination after education, the net change was not as significant as the degree of change in women’s awareness of HPV. The high acceptability of HPV vaccines have been reported both in developing [10, 16] and developed countries [29–31]. Our study results suggested that the attitudes toward HPV vaccination may not simply rely on women’s awareness of HPV. Simply educating women about HPV and HPV vaccines may not be sufficient to influence their attitudes, as attitudes may be driven by other, non-information-based preferences. As ‘I don’t think vaccination works’ and ‘I have no risk’ were listed as the two primary reasons for unwillingness to be vaccinated, more information about the efficacy of HPV vaccines and the HPV prevalence especially in high risk areas should be presented to women during the educational intervention in the future.

In order to guarantee the flow of the cervical cancer screening process, the baseline specific knowledge of HPV (e.g. the risk of HPV, its link between cervical cancer etc.) and its related diseases were not examined, however, the previous study indicated poor specific knowledge about HPV especially among rural Chinese women [10]. A study from the U.S has also reported that few women have heard of HPV, and did not always understand the link between cervical cancer and HPV despite the media oriented education campaign carried out prior to and after the availability of a quadrivalent prophylactic vaccine in the U.S in 2006 [32]. In some cases the participants were taken aback by the link between HPV and cervical cancer and had difficulty believing the link existed [32, 33]. Our study found that after the group education, more than 60 % of women knew that HPV is related to cervical cancer, whereas, only 5 % knew the relationship between HPV and genital warts, suggesting that intervention may be effective in increasing women’s specific knowledge of HPV related diseases, especially the most serious outcome of developing cervical cancer. More should be done to increase women’s knowledge regarding the link between HPV and genital warts if a widespread educational campaign is carried out in the future in mainland China.

Studies illustrated that greater knowledge about HPV may be associated with greater vaccine acceptability [31–39] and that poor knowledge about HPV and HPV vaccination have direct implications for women’s participation in cervical screening [7, 8]. Although the effectiveness of hospital based group education on HPV and cervical cancer awareness was observed in our study, continuous education that is incorporated into health-education from early childhood would be the most effective measure to help prevent cervical cancer [9, 40]. Some other educational intervention studies suggested that the efficacy of interventions depends on their duration to a large extent and also requires regular ‘booster’ sessions [9, 41, 42] On the other hand, when designing HPV-focused interventions, pooled efforts should be considered not only from the health care providers but also from the school teachers, the parents,the public media and the government to achieve effective results [43]. Advantages of such brief educational interventions include that they are inexpensive and could be easily conducted in the health care environment [44, 45], which make it more feasible and realistic in low resource settings where health care resources are limited.

There are several limitations of the study that need to be considered. The study was undertaken in one cervical cancer high risk rural county and may not represent the general Chinese population. The lack of a matched control group weakens the ability to assess the effectiveness of the reported intervention. In addition, the short term effectiveness does not necessarily guarantee a long term impact, and a follow up study with monthly intervals was not conducted to evaluate the duration of effect as well as to help decide the interval for a ‘booster’ session in future education campaigns. Finally, the strongest measure of the effectiveness of such an intervention would be associations with long-term outcomes such as decrease in cervical cancer incidence and mortality [9], however this was beyond the scope of our study.

One of the major strengths of the present study is that this is the first study to evaluate a simple method to a conduct hospital based group education to change women’s knowledge and attitudes toward HPV and its vaccines in China. Additionally, the relatively large sample size renders the results valid for assessing the short-term effectiveness of the reported program and useful for developing further educational interventions.

## Conclusions

In conclusion, this is the first intervention study on changing rural Chinese women’s knowledge and attitudes toward HPV and its vaccines. Our results confirm that a brief, hospital-based educational intervention is feasible to conduct in low resource settings and is appropriate in increasing awareness and acceptability, although the observed impact on attitudes towards prevention was less compared with the impact in HPV awareness. On the basis of our results, education campaigns, particularly targeting women with limited education and less access to public media or other educational channels are needed in the near future, to improve knowledge regarding HPV in the general population. A multi-center nation-wide randomized trial to test the intervention in both rural and urban Chinese women with follow-up evaluation would build upon and broaden the impact of this study.

## Abbreviations

HPV: Human Papillomavirus
LCMCCSS: Low-Cost Molecular Cervical Cancer Screening Study
PATH: Program for Appropriate Technology in Health
CICAMS: Cancer Institute of the Chinese Academy of Medical Sciences
CIN: Cervical Intraepithelial Neoplasia
